# High-risk sexual behaviors while on depot medroxyprogesterone acetate as compared to oral contraception

**DOI:** 10.1186/s40834-016-0035-0

**Published:** 2017-01-07

**Authors:** Deborah Bartz, Rie Maurer, Jessica Kremen, Jennifer M. Fortin, Elizabeth Janiak, Alisa B. Goldberg

**Affiliations:** 1 The Department of Obstetrics, Gynecology, and Reproductive Biology, Brigham and Women’s Hospital, 75 Francis St, Boston, MA 02115 USA; 2 Planned Parenthood League of Massachusetts, 1055 Commonwealth Ave, Boston, MA 02115 USA; 3 Center for Clinical Investigation, Brigham and Women’s Hospital, 75 Francis St, Boston, MA 02115 USA; 4 1620 Tremont St, OBC-3, Boston, MA 02120 USA

**Keywords:** Sexual behavior, Depot medroxyprogesterone acetate, Oral contraceptive pills, *Chlamydia*

## Abstract

**Background:**

Depot medroxyprogesterone acetate (DMPA) contraceptive use is associated with an increased risk for *Chlamydia* infection. However, prior studies inadequately account for potential differences in sexual behavior between users of DMPA and users of other contraceptive methods. In this study we compare sexual risk-taking behavior in women using DMPA to women using oral contraceptive pills (OCP) to assess risk of *Chlamydia trachomatis* infection.

**Methods:**

In this cross-sectional study of 630 reproductive-aged women seeking routine gynecologic care (449 OCP and 181 DMPA users) sexual risk-taking was evaluated by use of the Safe Sex Behavior Questionnaire, a validated measure of sexual behaviors and attitudes. All women were screened for *Chlamydia*. Logistic regression estimated the association of contraceptive choice, sexual behaviors, and *Chlamydia* infection.

**Results:**

Oral contraceptive pill users differed from DMPA users in age, race, marital status, education level, and pregnancy history (*p*-values all <0.05). Oral contraceptive pill users had used their method of contraception for longer average duration (*p* < 0.01) and reported greater frequency of condom use (*p* < 0.01). Eleven (2.5%) OCP and 2 (1.1%) DMPA users had *Chlamydia* (*p* = NS).

**Conclusions:**

Oral contraceptive pill and DMPA users differed with respect to both demographic factors and frequency of condom use. Odds of current *Chlamydia* infection did not differ between OCP and DMPA users when controlling for sexual risk-taking or demographic factors, though due to low *Chlamydia* rates in our population, this study was underpowered to detect this difference.

## Background

The injectable hormonal contraceptive depot medroxyprogesterone acetate (DMPA) is currently used by over 41 million women worldwide [1]. The ease of administration, high efficacy, lack of estrogen and duration of action make DMPA an attractive contraceptive for many women around the world.

*Chlamydia trachomatis* is the most commonly reported sexually transmitted infection (STI), and its incidence continues to rise by 3.3–4.9% per year for US women [2]. Three prospective human studies suggest that use of DMPA for contraception increases a woman’s risk for *Chlamydia* infection, with hazard ratios (HR) for *Chlamydia* infection reported as high as 3.6 when compared to oral contraceptive pill users [3, 4] or contraceptive non-users [3, 5]. The authors of these studies have primarily posited physiologic changes induced by exogenous hormone administration, such as changes in the immune system or vaginal and cervical epithelia, as the cause of higher *Chlamydia* acquisition among users of DMPA [3, 5]. However, follow up analysis and investigation of functional change of cervical ectopy [3, 5, 6], vaginal pH [5], or cervicovaginal flora [7] have not demonstrated a difference in DMPA users as compared to non-hormonal contraception users.

These previous studies that have focused on a physiologic mechanism to explain the association of DMPA use and *Chlamydia* acquisition have inadequately controlled for sexual behaviors that may put DMPA users at risk for sexually transmitted infections [3–5]. Depot medroxyprogesterone acetate users may differ from users of other methods or from non-contraceptors because DMPA is a simple-to-use method well-suited to women with busy schedules, chaotic lives, or intermittent access to health care. These life characteristics may be associated with higher-risk sexual behavior.

We therefore sought to investigate whether the apparent association between use of DMPA and heightened risk for *Chlamydia* acquisition could be an artifact of uncontrolled confounding variables, particularly sexual risk-taking. We chose to compare DMPA users to oral contraceptive pill (OCP) users because both methods are reversible hormonal methods that afford excellent protection against pregnancy, no protection against *Chlamydia* acquisition, and both are frequently used by young women, a population at risk for *Chlamydia* infection [8].

## Methods

Consecutive women presenting for routine gynecologic care at a high-volume, urban reproductive health clinic were recruited to participate if they met the following criteria: were 18–49 years old, had initiated use of either DMPA or OCPs at least 1 week prior to the study visit, and could speak and read either English or Spanish. Women who were pregnant, had a prior total hysterectomy, or a recent cervical procedure (such as a colposcopically directed biopsy or cone), a potential non-behavioral risk factor for cervical infection, were ineligible. Participants may or may not have been presenting for STI testing as part of their care; the reason for the visit of all subjects was recorded. We presumed a *Chlamydia* prevalence of 13% in DMPA users versus 5% in OCP users based on existing national data from similar clinical settings [9], estimated that we would be able to recruit at a 1:2 ratio into the DMPA and OCP arms, respectively, and calculated that we would need at least 675 subjects (225 in the DMPA arm and 450 in the OCP arm) to detect a difference of 8% in the prevalence of *Chlamydia* between DMPA and OCP users with 90% power and two-sided alpha of 0.05% and assuming a 10% study noncompliance rate. Subject consent to participate was given verbally in order to maintain complete anonymity given the sensitive nature of the behavioral data collected. The study received approval from Partners Healthcare Human Research Committee IRB.

Upon enrollment, participants completed a 47-item paper, self-administered survey, half of which collected information regarding sociodemographics, obstetric, gynecologic and STI history, STI knowledge, contraceptive method history, and condom use. The second half of the survey contained 24 questions from the Safe Sex Behavior Questionnaire (SSBQ), a validated measure of sexual behaviors and attitudes designed to assess level of sexual risk-taking [10]. The responses for this 24-item survey are all given on a 4-point Likert scale, resulting in a summed risk-taking score ranging from 24 to 96, with a higher score suggestive of safer, lower risk-taking sexual behavior.

Participants provided a urine specimen to detect *Chlamydia trachomatis* through nucleic acid amplification, with results reported as binary positive or negative [11]. These urine specimens, labeled anonymously by study number only, were collected, stored, transported and analyzed in the exact manner as all *Chlamydia* tests performed in this clinic outside of the study during this same time period [12]. At enrollment, all participants were given a copy of their personal study number and a phone number to call a study nurse for *Chlamydia* results. This was the only way subjects could access their anonymous study results; those patients who did not call study nurse with their study number did not receive their culture results. Subjects who called and were found to be positive were required to identify themselves over the phone in order to be prescribed antibiotic therapy. Subjects who desired or had clinical indications for routine STI testing and follow-up by clinical staff had been offered additional testing under normal procedures at the clinic on the day of their visit before study enrollment. Therefore, some subjects had two *Chlamydia *cultures sent, one from their clinic visit and one from the study protocol. The IRB approved these *Chlamydia* reporting procedures.

We compared numerical variables, including the summated SSBQ score, between the two birth control groups using either t-tests or Wilcoxon Rank-Sum tests. No pattern in missing SSBQ responses was apparent and because omissions appeared to be randomly distributed in the sample, median substitution method was used for the analysis where applicable.

We compared categorical variables using Chi-square or Fisher’s exact tests. *Chlamydia* infection rates were reported with exact 95% confidence intervals. We performed logistic regression to assess the relationship between contraceptive method and *Chlamydia* infection adjusting for potential confounding variables. *P*-values and odds ratios with 95% confidence intervals are reported. All analyses were performed with SAS v9.2 statistical software (SAS Institute, Cary, NC).

## Results

Over the course of 12 months of May 2007 to May 2008 we approached 1,869 patients. Nine hundred thirty-five were not current OCP or DMPA users and thus, did not meet eligibility. Six hundred thirty-one of 934 eligible patients elected to participate, for a participation rate of 67.6%. The study was closed before full recruitment of the DMPA exposure arm was completed (225 intended participants) because the lower than expected *Chlamydia* rates would require an unfeasibly large sample size to detect a difference between groups. One OCP participant who did not complete half of the survey data pertaining to sexual risk-taking was excluded from analysis; the final analytic sample includes 630 study participants, 449 OCP and 181 DMPA users. Overall response rate for each question was very high with fewer than 3% of responses missing for all scale items except one, “I avoid sexual intercourse when I have sores or irritation in my genital area” (9.8% missing).

Demographic characteristics of OCP and DMPA users are summarized in Table 1; the two exposure arms differed in all demographic measures. The overall mean sexual risk-taking score as assessed by the SSBQ was not significantly different between subjects using OCPs (55.7 ± 6.9) versus DMPA (54.5 ± 7.8) (*p* = 0.09). Table 2 summarizes associations between contraceptive method and selected items on the SSBQ. Oral contraceptive users reported longer duration of current contraceptive method use (*p* < 0.01) and greater condom use (*p* < 0.01). Age of first sex was found to be earlier in DMPA users (*p* < 0.01) and fewer DMPA users co-habited with their current partners compared to OCP users (*p* < 0.01). Oral contraceptive users more frequently stated that their primary reason for visiting the clinic at the time of study enrollment was to get tested for STIs due to symptoms or suspected exposure (*p* < 0.01). Subjects who presented for STI testing in either group were more likely to test positive for *Chlamydia* (*p* < 0.01). However, the relationship between contraceptive method and *Chlamydia* infection remained non-significant after adjusting for the reason for the visit.

**Table 1:** Baseline subject characteristics by birth control method used (*n* = 630)

Characteristic	OCP Users (*n* = 449)	DMPA Users (*n* = 181)	*P* value
Age in years, mean [range]	23 [21, 26]	24 [22, 27]	0.04
Race			<0.01
White/European American	347 (77.3)	103 (56.9)	
Black/African American	19 (4.2)	38 (21.0)	
Latina	23 (5.1)	24 (13.3)	
Other	60 (13.4)	16 (8.8)	
Marital Status			<0.01
Single	201 (44.8)	59 (32.6)	
Married	15 (3.3)	15 (8.3)	
Divorced	2 (0.5)	3 (1.7)	
In a relationship	231 (51.5)	104 (57.5)	
Education			<0.01
High school or less	24 (5.4)	29 (16.0)	
Some college	133 (29.6)	72 (39.8)	
College graduate	206 (46.0)	54 (29.8)	
Graduate or professional school	86 (19.2)	26 (14.4)	
Income (personal, annual)			<0.01
$9,999 or less	187 (41.8)	50 (27.8)	
$10,000–34,999	162 (36.2)	80 (44.4)	
$35,000 or more	98 (21.9)	50 (27.8)	
Number of Prior Pregnancies Mean [range]	0 [0, 0]	1 [0, 2]	<0.01
Any prior Pregnancy	89 (19.9)	92 (50.8)	<0.01

Categorical variables are presented with frequency counts (%). Numerical variables are presented with mean [range] as noted

**Table 2:** Selected Safe Sex Behavioral Questionnaire responses by birth control method used (*n* = 630)

Question	OCP Users (*n* = 449)	DMPA Users (*n* = 181)	*P* value
Condom use:			<0.01
Never or 0% of the time	43 (9.6)	30 (16.7)	
Vary rarely-25% of the time	129 (28.7)	65 (36.3)	
25-75% of the time	140 (31.2)	40 (22.4)	
Almost always -100% of the time	137 (30.5)	44 (24.6)	
When did you start using current BC method?			<0.01
Less than 12 months ago	110 (24.5)	77 (42.8)	
12–23 months ago	92 (20.5)	31 (17.2)	
24–48 months ago	96 (21.4)	39 (21.7)	
More than 48 months ago	151 (33.6)	33 (18.3)	
Sexually Transmitted Infection			0.88
Have you ever been diagnosed with a sexually transmitted infection?			
Yes	75 (16.7)	31 (17.2)	
No	374 (83.3)	150 (82.8)	
How old were you when you had sex very first time?	17.5 ± 2.2	16.9 ± 2.3	<0.01
How many men have you had sex with?	6 [3, 11]	6 [3, 10]	0.82
Are you monogamous?			0.28
Yes	365 (81.5)	157 (86.7)	
No	37 (8.2)	11 (6.1)	
Not sexually active	46 (10.3)	13 (7.2)	
Are condoms effective?			0.72
Not at all	6 (1.4)	4 (2.2)	
Somewhat	151 (33.6)	61 (33.9)	
Very	292 (65.0)	115 (63.9)	
Have you ever been treated for a sexually transmitted infection?			0.69
Yes	5 (1.1)	3 (1.7)	
No	444 (98.9)	178 (98.3)	
Are you currently living with a sexual partner?			<0.01
Yes	133 (30.0)	80 (44.2)	
No	316 (70.4)	101 (55.8)	
Reason for appointment			<0.01
Vaginal itching/STI	136 (30.3)	10 (5.5)	

Categorical variables are presented with frequency counts (%). Numerical variables are presented with mean ± SD or median [Q1, Q3]

Eleven OCP users (2.5%, 95% CI: 1.4–4.3%) and two DMPA users (1.1%, 95% CI: 0.3–3.9%) tested positive for *Chlamydia* (*p* = NS). The mean sexual risk-taking score was not statistically different between subjects with (53.9 ± 6.9) and without (55.4 ± 7.2) *Chlamydia* infection. The lack of significant difference in the odds of *Chlamydia* infection according to contraceptive method persisted after adjusting for sexual risk-taking scores. Lastly, there was no significant association between the frequency of *Chlamydia* infection and contraceptive method after adjusting for race and marital status.

## Discussion

We sought to assess whether women who chose a short-term, high-maintenance contraceptive method, the daily OCP, differ in risk-taking behavior from women who chose the long-acting, low-maintenance DMPA injection. We found that DMPA and OCP users did differ in both sexual risk-taking behaviors and in demographics, such as relationship status, that may influence *Chlamdyia* acquisition risk. However, due to unexpectedly low *Chlamydia* infection rates within our population, this study was underpowered to detect a difference in infection prevalence.

The hypothesized physiological pathway for an association between DMPA and *Chlamydia* is challenged by other investigations that have found a possible protective effect [13] or no effect [14, 15] of hormonal contraception use on risk of *Chlamydia* acquisition. Furthermore, while a recent meta-analysis demonstrated an increased acquisition of another sexually transmitted infection, HIV, among DMPA users, the evidence suggests that there is a behavioral component to this risk. The risk of HIV was higher in all women who use DMPA (pooled HR 1.40, 95% CI 1.16–1.69) compared with use of non-hormonal or no method. However, this effect was attenuated when analysis was restricted to the eight studies recruiting from the general population (pooled HR 1.31, 95% CI 1.10–1.57), excluding studies from populations with high-risk sexual lifestyles such as commercial sex-workers [16]. Estimates for HIV risk with DMPA use were higher from studies of women with high-risk lifestyles (HR 1.73, 95% CI 1.28–2.34 [17] and HR 3.93 1.37–11.2 [18]).

Our low *Chlamydia* rate may in part reflect bias introduced by self-selection into the study population. While we took measures to reassure patients of the anonymous nature of data collection, given the sensitive nature of the sexual behavior questions that were queried and the resulting patient-directed *Chlamydia* result reporting, it is possible that women who considered themselves to be at higher risk for infection chose not to participate, biasing our sample towards a non-infected population. Since our study inclusion criteria excluded patients initiating a new contraceptive method, it is also possible that we recruited women who had recently started their contraceptive method at the clinic and who had been previously screened, diagnosed, and treated for *Chlamydia* as part of a relatively recent contraception initiation visit.

The reliability and validity of patient-reported sexual history data has been studied with conflicting results [19, 20]. Our use of a validated survey instrument and anonymous study design was intended to minimize reporting bias and promote accuracy of behavioral self-reports. However, only a randomized controlled trial of contraceptive methods would be sufficient to resolve all potential behavioral confounders in exploring the relationship between hormonal contraception and STI acquisition, including reporting bias that is potentially differential with respect to predictors of interest. Several studies have randomized subjects to hormonal versus non-hormonal contraception and found discontinuation and pregnancy rates similar to the general population using these contraceptive methods [21–23], discounting concerns that randomization to contraceptive method is unethical. Furthermore, Hubacher and colleagues [24] conducted a cross-sectional survey to assess the feasibility of randomizing women to an intrauterine device or DMPA in order to assess STI risk in DMPA users, and found that 70% of respondents stated they would accept randomization into one of these treatment arms.

## Conclusion

Depot medroxyprogesterone acetate is an important contraceptive method for many women around the world. Our findings suggest that women who choose DMPA may have behavioral risk factors that increase their risk for STI’s, however, composite sexual risk-taking scores did not differ between DMPA and OCP users. This study was underpowered to detect a difference in Chlamydia rates between users of these two contraceptive methods. Interventions should be directed towards improved safe-sex behavior amongst DMPA users. Experts at the World Health Organization have recently reviewed the data on DMPA and STI risk and agree that prior work suggesting an association between progesterone-only injectable contraception and STI acquisition have important methodological limitations that hinder interpretation, that DMPA is still a good method of contraception for women, and that instead of directing patients away from this method in order to decrease STI risk, clinicians should promote STI preventative measures, such as male and female condoms among DMPA users [25].

## Abbreviations

DMPA: Depot medroxyprogesterone acetate
HR: Hazard ratio
OCP: Oral contraceptive pills
SSBQ: Safe sex behavior questionnaire
STI: Sexually transmitted infection
